# Hexacarbonyl­technetium(I) perchlorate

**DOI:** 10.1107/S1600536808025208

**Published:** 2008-08-09

**Authors:** V. V. Gurzhiy, A. E. Miroslavov, G. V. Sidorenko, A. A. Lumpov, S. V. Krivovichev, D. N. Suglobov

**Affiliations:** a St Petersburg State University, Universitetskaya nab. 7/9, 199034 St Petersburg, Russian Federation; b Khlopin Radium Institute, Research and Production Association, 2-nd Murinskii pr. 28, 194021 St Petersburg, Russian Federation

## Abstract

The title compound, [Tc(CO)_6_]ClO_4_, was synthesized by the reaction of [TcCl(CO)_5_] with AgClO_4_, followed by acidification with HClO_4_ under a CO atmosphere. The [Tc(CO)_6_]^+^ cation has close to idealized octa­hedral geometry, with the bond angles between *cis*-CO groups close to 90° and the Tc—C bond lengths in the range 2.025 (3)–2.029 (3)Å. The perchlorate anion is disordered over two crystallographically equivalent half-occupied positions. The Tc atom in the [Tc(CO)_6_]^+^ cation is located on an inversion centre.

## Related literature

For the first report on the [Tc(CO)_6_]^+^ cation, see: Hieber *et al.* (1965 ▶). For related literature, see: Aebischer *et al.* (2000 ▶); Alberto *et al.* (1996 ▶, 1998 ▶); Baturin *et al.* (1994*a* ▶,*b* ▶); Grigor’ev *et al.* (1997*a* ▶,*b* ▶); Miroslavov *et al.* (2008*a* ▶,*b* ▶); Schwochau (2000 ▶).
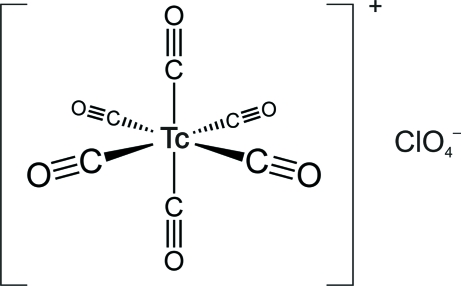


## Experimental

### 

#### Crystal data

[Tc(CO)_6_]ClO_4_
*M* *_r_* = 366.42
Monoclinic, 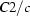

*a* = 13.227 (4) Å
*b* = 6.8002 (18) Å
*c* = 13.616 (3) Å
β = 112.56 (2)°
*V* = 1131.0 (5) Å^3^
*Z* = 4
Mo *K*α radiation
μ = 1.55 mm^−1^
*T* = 293 (2) K
0.20 × 0.18 × 0.10 mm

#### Data collection

Stoe IPDS-2 diffractometer
Absorption correction: integration (*X-RED* and *X-SHAPE*; Stoe & Cie, 2005 ▶) *T*_min_ = 0.620, *T*_max_ = 0.723
4935 measured reflections
1508 independent reflections
1224 reflections with *I* > 2σ(*I*)
*R*_int_ = 0.035

#### Refinement

*R*[*F* ^2^ > 2σ(*F* ^2^)] = 0.030
*wR*(*F* ^2^) = 0.067
*S* = 1.06
1508 reflections
99 parameters
Δρ_max_ = 0.32 e Å^−3^
Δρ_min_ = −0.44 e Å^−3^

### 

Data collection: *X-AREA* (Stoe & Cie, 2007 ▶); cell refinement: *X-AREA*; data reduction: *X-RED* (Stoe & Cie, 2005 ▶); program(s) used to solve structure: *SHELXL97* (Sheldrick, 2008 ▶); program(s) used to refine structure: *SHELXL97* (Sheldrick, 2008 ▶); molecular graphics: *ATOMS* (Dowty, 2000 ▶); software used to prepare material for publication: *publCIF* (Westrip, 2008 ▶).

## Supplementary Material

Crystal structure: contains datablocks global, I. DOI: 10.1107/S1600536808025208/fj2134sup1.cif

Structure factors: contains datablocks I. DOI: 10.1107/S1600536808025208/fj2134Isup2.hkl

Additional supplementary materials:  crystallographic information; 3D view; checkCIF report

## Figures and Tables

**Fig. 1.**
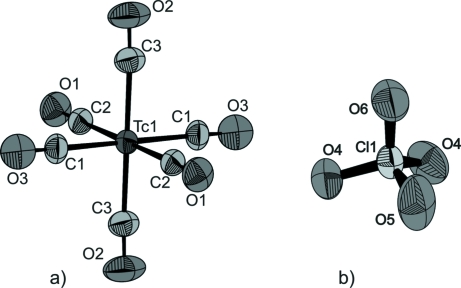
View of the [Tc(CO)6]+cation (a) and one component of the disordered perchlorate anion (b) in the structure of [Tc(CO)6]ClO4. Thermal ellipsoids are drawn at the 50% probability level.

**Fig. 2.**
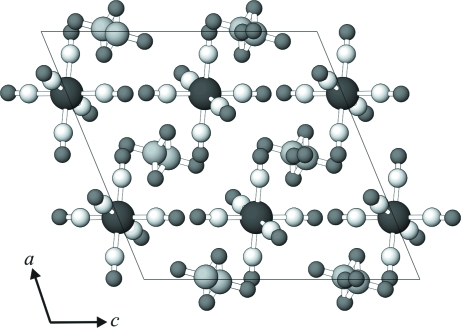
Crystal structure of [Tc(CO)6]ClO4. Tc atoms are dark-grey, O atoms are grey, Cl atoms are light-grey, and C atoms are white circles.

**Fig. 3.**
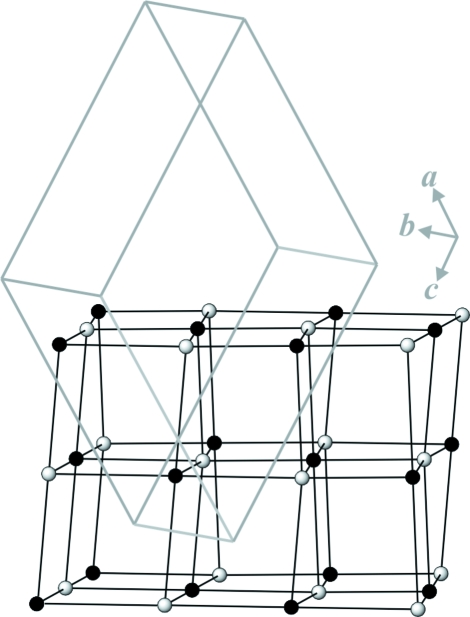
Relationship of title structure to NaCl structure.

## supplementary crystallographic information

### Comment

Among technetium(I) carbonyl complexes, the highest carbonyl, [Tc(CO)_6_]^+^ cation, is the least studied compared to penta-, tetra-, and especially tricarbonyl complexes (Schwochau, 2000). No data on the crystal structure of its salts are available. More detailed study of this cation is significant for the development of the coordination chemistry of technetium (and d block as a whole). The first report on the [Tc(CO)_6_]^+^ cation is dated by Hieber *et al., 1965* prepared this species in the form of the solid compound [Tc(CO)_6_][AlCl_4_] by the solid-phase reaction of [TcCl(CO)_5_] with AlCl_3_ under high CO pressure (300 atm) at 363 K (Heiber *et al.*, 1965). The product was characterized by chemical analysis, and the cation appeared to be stable in solutions. Relatively recently (Aebischer *et al.*, 2000) observed successive formation of higher technetium carbonyls [Tc(CO)_n_(H_2_O)_6–n_]^+^ (n = 4–6) in aqueous solution (2 *M* HClO_4_) from the complex [Tc(CO)_3_(H_2_O)_3_]^+^ at room temperature and moderately high CO pressure (about 50 atm), after removal of chloride ions. The reaction progress was monitored by the ^99^Tc and ^13^C NMR. The relative content of the [Tc(CO)_6_]^+^ cation in the mixture of technetium carbonyl species was low, and no solid salt of this cation was isolated. Here we report on the synthesis and crystal structure of hexacarbonyltechnetium(I) perchlorate, [Tc(CO)_6_]ClO_4_.

### Experimental

Pentacarbonyltechnetium chloride [TcCl(CO)_5_] (SU Inventor's Certificate 1512003) was dissolved in boiled water, and a stoichiometric amount of AgClO_4_ was added after cooling to remove chloride ions interfering with the synthesis (Miroslavov *et al.*, 2008*a*). The resulting solution was acidified with HClO_4_ to a concentration of 2 *M* and treated with CO in a pressure vessel (443 K, 150 atm, 1 h). After completing the reaction and removing the excess of CO, the reaction system consisted of an aqueous solution and a colorless crystalline precipitate. The precipitate was separated, washed with water and methylene chloride (to remove an impurity of [TcCl(CO)_5_] (Miroslavov *et al.*, 2008*a*)), and dried in air. The product was identified as [Tc(CO)_6_]ClO_4_. Some of the crystals appeared to be suitable for an X-ray diffraction analysis. ^99^Tc NMR(CH_3_OH): -1924 p.p.m.. IR (CH_3_CN): n_CO_ 2095 cm^-1^. Found Tc, %: 27.12. C_6_ClO_10_Tc. Calculated Tc, %: 27.01. The IR spectrum was recorded on a Shimadzu FTIR 8700 spectrophotometer. The ^99^Tc NMR spectrum was taken on a Bruker WP-200 spectrometer.

### Refinement

The crystal structure of [Tc(CO)_6_]ClO_4_ contains one symmetrically independent Tc^+^ cation octahedrally coordinated by six carbon atoms (Figs. 1, 2). The Tc—C=O fragments are linear to within 3°. The coordination polyhedron of technetium in the [Tc(CO)_6_]^+^ cation is close to an ideal octahedron, with the bond angles between *cis*-CO groups equal to 90° (within ±1.5°) and the Tc–C bond lengths in the range of 2.025–2.029 Å. These distances are significantly (by 0.1–0.15 Å) longer than the Tc–C distances in *trans*-OC–Tc–σ donor fragments, *e.g.*: [TcI(CO)_5_](Tc–C*_trans_*_-I_) 1.938 (Grigor'ev *et al.*, 1997*a*), [TcI(CO)_4_]_2_ (Tc–C*_trans_*_-I_) 1.89–1.92 (Grigor'ev *et al.*, 1997*b*), [TcCl(CO)_3_]_4_ 1.903 (Baturin *et al.*, 1994*a*), [TcBr(CO)_3_(en)] 1.882–1.889 (Baturin *et al.*, 1994*b*), [Tc(OH)(CO)_3_]_4_ 1.886–1.905 Å (Alberto *et al.*, 1998). At the same time, they are only slightly longer than the Tc–C distances in *trans*-OC–Tc–π acceptor fragments of other structurally examined complexes (π acceptor is another CO group, PPh_3_, or Bu*^t^*NC): [Tc(CO)_5_(Bu*^t^*NC)]ClO_4_ 1.999–2.022 (Miroslavov *et al.*, 2008*b*), [Tc(CO)_5_(PPh_3_)]CF_3_SO_3_ 1.985–2.019 (Alberto *et al.*, 1998) (in these two compounds, the lengths of the equatorial and axial Tc–CO bonds are similar), [*fac*-Tc(CO)_3_(Bu*^t^*NC)_3_]NO_3_ 1.963–1.975 (Alberto *et al.*, 1996) [TcI(CO)_5_] (Tc–C~trans-CO~) 2.015 (Grigor'ev *et al.*, 1997*a*), [TcI(CO)_4_]_2_ (Tc–C*_trans_*_-CO_) 1.98–2.01 Å (Grigor'ev *et al.*, 1997*b*). The large difference between the Tc–CO bond lengths in cases when the transposition to the CO group is occupied by a π acceptor or a σ donor can be attributed to the *trans* effect (competition between the π acceptors arranged *trans* to each other for the same occupied d orbital of the metal ion). A certain *cis* effect, however, also takes place, because the Tc–CO bonds in the [Tc(CO)_6_]^+^ cation are somewhat longer than the Tc–CO bonds in *trans*-OC–Tc–CO fragments of complexes containing in *cis* positions ligands that are σ donors or π acceptors weaker than CO.

The Cl atom in the structure of [Tc(CO)_6_]ClO_4_ is tetrahedrally coordinated by four O atoms (mean Cl–O distance is 1.403 Å). The perchlorate anion is disordered over two crystallographically equivalent half-occupied positions (Fig. 2) with the total site-occupation factor (s.o.f.) equal to 1.0. The central atoms of [Tc(CO)_6_]^+^ octahedra and [ClO_4_]^-^ tetrahedra (Tc and Cl respectively) form a distorted NaCl-type lattice oriented along *a*_NaCl_ [110], *b*_NaCl_ [10–1], *c*_NaCl_ [-110] (Fig. 3).

### Crystal data

**Table:**

[Tc(CO)_6_]ClO_4_	*F*_000_ = 704
*M**_r_* = 366.42	*D*_x_ = 2.152 Mg m^−^^3^
Monoclinic, *C*2/*c*	Mo *K*α radiation λ = 0.71073 Å
Hall symbol: -C 2yc	Cell parameters from 5446 reflections
*a* = 13.227 (4) Å	θ = 2.0–29.6º
*b* = 6.8002 (18) Å	µ = 1.55 mm^−^^1^
*c* = 13.616 (3) Å	*T* = 293 (2) K
β = 112.56 (2)º	Prism, colorless
*V* = 1131.0 (5) Å^3^	0.20 × 0.18 × 0.10 mm
*Z* = 4	

### Data collection

**Table:**

Stoe IPDS-2 diffractometer	1508 independent reflections
Radiation source: fine-focus sealed tube	1224 reflections with *I* > 2σ(*I*)
Monochromator: graphite	*R*_int_ = 0.035
Detector resolution: 6.67 pixels mm^-1^	θ_max_ = 29.2º
*T* = 293(2) K	θ_min_ = 3.2º
rotation method scans	*h* = −18→18
Absorption correction: integration(X-RED and X-SHAPE; Stoe & Cie, 2005)	*k* = −9→8
*T*_min_ = 0.620, *T*_max_ = 0.723	*l* = −18→18
4935 measured reflections	

### Refinement

**Table:**

Refinement on *F*^2^	Secondary atom site location: difference Fourier map
Least-squares matrix: full	*w* = 1/[σ^2^(*F*_o_^2^) + (0.0289*P*)^2^ + 1.543*P*] where *P* = (*F*_o_^2^ + 2*F*_c_^2^)/3
*R*[*F*^2^ > 2σ(*F*^2^)] = 0.031	(Δ/σ)_max_ < 0.001
*wR*(*F*^2^) = 0.067	Δρ_max_ = 0.32 e Å^−^^3^
*S* = 1.06	Δρ_min_ = −0.44 e Å^−^^3^
1508 reflections	Extinction correction: SHELXL97 (Sheldrick, 2008), Fc^*^=kFc[1+0.001xFc^2^λ^3^/sin(2θ)]^-1/4^
99 parameters	Extinction coefficient: 0.0043 (12)
Primary atom site location: structure-invariant direct methods	

### Special details

**Table:**

Geometry. All e.s.d.'s (except the e.s.d. in the dihedral angle between two l.s. planes) are estimated using the full covariance matrix. The cell e.s.d.'s are taken into account individually in the estimation of e.s.d.'s in distances, angles and torsion angles; correlations between e.s.d.'s in cell parameters are only used when they are defined by crystal symmetry. An approximate (isotropic) treatment of cell e.s.d.'s is used for estimating e.s.d.'s involving l.s. planes.
Refinement. Refinement of *F*^2^ against ALL reflections. The weighted *R*-factor *wR* and goodness of fit *S* are based on *F*^2^, conventional *R*-factors *R* are based on *F*, with *F* set to zero for negative *F*^2^. The threshold expression of *F*^2^ > σ(*F*^2^) is used only for calculating *R*-factors(gt) *etc*. and is not relevant to the choice of reflections for refinement. *R*-factors based on *F*^2^ are statistically about twice as large as those based on *F*, and *R*- factors based on ALL data will be even larger.

### Fractional atomic coordinates and isotropic or equivalent isotropic displacement parameters (Å^2^)

**Table:**

	*x*	*y*	*z*	*U*_iso_*/*U*_eq_	Occ. (<1)
Tc1	0.2500	0.2500	0.0000	0.03673 (14)	
C1	0.2509 (2)	0.1350 (5)	0.1375 (2)	0.0484 (7)	
C2	0.3021 (2)	−0.0119 (5)	−0.0345 (2)	0.0446 (6)	
C3	0.4088 (2)	0.3328 (5)	0.0721 (3)	0.0501 (7)	
O1	0.3312 (2)	−0.1557 (4)	−0.0529 (2)	0.0632 (6)	
O2	0.49658 (19)	0.3762 (4)	0.1078 (3)	0.0774 (8)	
O3	0.2481 (2)	0.0685 (5)	0.2111 (2)	0.0778 (8)	
O4	0.0932 (2)	−0.2268 (5)	−0.1933 (3)	0.0814 (9)	
O5	0.503 (3)	0.0179 (9)	0.2724 (16)	0.109 (7)	0.50
O6	0.5401 (5)	−0.1423 (12)	0.1398 (5)	0.0856 (18)	0.50
Cl1	0.50996 (15)	−0.1697 (2)	0.22853 (14)	0.0480 (4)	0.50

### Atomic displacement parameters (Å^2^)

**Table:**

	*U*^11^	*U*^22^	*U*^33^	*U*^12^	*U*^13^	*U*^23^
Tc1	0.03421 (17)	0.04318 (19)	0.03316 (17)	0.00255 (14)	0.01331 (11)	−0.00340 (15)
C1	0.0481 (15)	0.0592 (18)	0.0423 (16)	0.0126 (13)	0.0222 (12)	0.0041 (13)
C2	0.0403 (13)	0.0519 (15)	0.0404 (14)	0.0037 (12)	0.0141 (11)	−0.0028 (12)
C3	0.0418 (14)	0.0527 (15)	0.0545 (18)	0.0005 (13)	0.0172 (13)	−0.0074 (14)
O1	0.0693 (15)	0.0567 (14)	0.0658 (16)	0.0148 (12)	0.0284 (12)	−0.0076 (12)
O2	0.0433 (12)	0.0782 (18)	0.105 (2)	−0.0091 (12)	0.0216 (13)	−0.0225 (16)
O3	0.0830 (18)	0.105 (2)	0.0609 (16)	0.0297 (16)	0.0444 (14)	0.0249 (16)
O4	0.0566 (14)	0.096 (2)	0.087 (2)	−0.0202 (14)	0.0229 (14)	−0.0261 (16)
O5	0.077 (4)	0.074 (3)	0.155 (19)	−0.002 (8)	0.021 (12)	−0.052 (8)
O6	0.072 (3)	0.120 (5)	0.061 (3)	−0.019 (3)	0.021 (3)	0.019 (4)
Cl1	0.0366 (7)	0.0512 (6)	0.0497 (12)	−0.0023 (6)	0.0094 (6)	−0.0015 (6)

### Geometric parameters (Å, °)

**Table:**

Tc1—C1^i^	2.025 (3)	O5—O5^iv^	0.59 (4)
Tc1—C1	2.025 (3)	O5—Cl1^iv^	1.286 (8)
Tc1—C3^i^	2.027 (3)	O5—Cl1	1.428 (11)
Tc1—C3	2.027 (3)	O6—Cl1	1.422 (6)
Tc1—C2^i^	2.029 (3)	O6—Cl1^iv^	2.144 (6)
Tc1—C2	2.029 (3)	Cl1—Cl1^iv^	0.728 (3)
C1—O3	1.113 (4)	Cl1—O5^iv^	1.286 (8)
C2—O1	1.114 (4)	Cl1—O4^v^	1.393 (3)
C3—O2	1.113 (4)	Cl1—O4^iii^	1.444 (3)
O4—Cl1^ii^	1.393 (3)	Cl1—O6^iv^	2.144 (6)
O4—Cl1^iii^	1.444 (3)		
			
C1^i^—Tc1—C1	180.0 (2)	O1—C2—Tc1	179.6 (3)
C1^i^—Tc1—C3^i^	91.27 (14)	O2—C3—Tc1	177.0 (3)
C1—Tc1—C3^i^	88.73 (14)	O5^iv^—Cl1—O4^v^	124.7 (11)
C1^i^—Tc1—C3	88.73 (14)	O4^v^—Cl1—O5	107.0 (10)
C1—Tc1—C3	91.27 (14)	O5^iv^—Cl1—O6	86.5 (11)
C3^i^—Tc1—C3	180.0 (3)	O4^v^—Cl1—O6	108.6 (3)
C1^i^—Tc1—C2^i^	89.61 (12)	O5—Cl1—O6	108.9 (9)
C1—Tc1—C2^i^	90.39 (12)	O5^iv^—Cl1—O4^iii^	112.3 (14)
C3^i^—Tc1—C2^i^	88.56 (12)	O4^v^—Cl1—O4^iii^	112.1 (3)
C3—Tc1—C2^i^	91.44 (12)	O5—Cl1—O4^iii^	111.5 (13)
C1^i^—Tc1—C2	90.39 (12)	O6—Cl1—O4^iii^	108.6 (3)
C1—Tc1—C2	89.61 (12)	O5^iv^—Cl1—O6^iv^	80.9 (11)
C3^i^—Tc1—C2	91.44 (12)	O4^v^—Cl1—O6^iv^	79.2 (2)
C3—Tc1—C2	88.56 (12)	O5—Cl1—O6^iv^	58.6 (10)
C2^i^—Tc1—C2	180.00 (17)	O6—Cl1—O6^iv^	167.4 (6)
O3—C1—Tc1	177.6 (3)	O4^iii^—Cl1—O6^iv^	76.4 (2)

Symmetry codes: (i) −*x*+1/2, −*y*+1/2, −*z*; (ii) *x*−1/2, −*y*−1/2, *z*−1/2; (iii) −*x*+1/2, −*y*−1/2, −*z*; (iv) −*x*+1, *y*, −*z*+1/2; (v) *x*+1/2, −*y*−1/2, *z*+1/2.

## References

[bb1] Aebischer, N., Schibli, R., Alberto, R. & Merbach, A. E. (2000). *Angew. Chem. Int. Ed.* **39**, 254–256. 10.1002/(sici)1521-3773(20000103)39:1<254::aid-anie254>3.0.co;2-f 10649393

[bb2] Alberto, R., Schibli, R., Egli, A., Abram, U., Abram, S., Kaden, T. A. & Schubiger, P. A. (1998). *Polyhedron*, **17**, 1133–1140. 10.1021/ic980112f 11670435

[bb3] Alberto, R., Schibli, R., Schubiger, P. A., Abram, U. & Kaden, T. A. (1996). *Polyhedron*, **15**, 1079–1089.

[bb4] Baturin, N. A., Grigor’ev, M. S., Kryuchkov, S. V., Miroslavov, A. E., Sidorenko, G. V. & Suglobov, D. N. (1994*a*). *Radiochemistry*, **36**, 199–201.

[bb5] Baturin, N. A., Grigor’ev, M. S., Kryuchkov, S. V., Miroslavov, A. E., Sidorenko, G. V. & Suglobov, D. N. (1994*b*). *Radiochemistry*, **36**, 202–204.

[bb6] Dowty, E. (2000). *ATOMS* Shape Software, Kingsport, Tennessee, USA.

[bb7] Grigor’ev, M. S., Miroslavov, A. E., Sidorenko, G. V. & Suglobov, D. N. (1997*a*). *Radiochemistry*, **39**, 204–206.

[bb8] Grigor’ev, M. S., Miroslavov, A. E., Sidorenko, G. V. & Suglobov, D. N. (1997*b*). *Radiochemistry*, **39**, 207–209.

[bb9] Hieber, W., Lux, F. & Herget, C. Z. (1965). *Naturforsch. Teil B*, **20**, 1159–1165.

[bb10] Miroslavov, A. E., Levitskaya, E. M., Sidorenko, G. V., Lumpov, A. A., Suglobov, D. N., Gurzhiy, V. V. & Krivovichev, S. V. (2008*a*). *Radiochemistry*, **50** In the press.

[bb11] Miroslavov, A. E., Lumpov, A. A., Sidorenko, G. V., Levitskaya, E. M., Gorshkov, N. I., Suglobov, D. N., Alberto, R., Braband, H., Gurzhiy, V. V., Krivovichev, S. V. & Tananaev, I. G. (2008*b*). *J. Organomet. Chem.* **693**, 4–10.

[bb12] Schwochau, K. (2000). *Technetium, Chemistry and Radiopharmaceutical Applications* New York: Wiley-VCH.

[bb13] Sheldrick, G. M. (2008). *Acta Cryst.* A**64**, 112–122. 10.1107/S0108767307043930 18156677

[bb14] Stoe & Cie (2005). *X-RED* and *X-SHAPE* Stoe & Cie GmbH, Darmstadt, Germany.

[bb15] Stoe & Cie (2007). *X-AREA* Stoe & Cie GmbH, Darmstadt, Germany.

[bb16] Westrip, S. P. (2008). *publCIF* In preparation.

